# Bilayer and three dimensional conductive network composed by SnCl_2_ reduced rGO with CNTs and GO applied in transparent conductive films

**DOI:** 10.1038/s41598-021-89305-1

**Published:** 2021-05-10

**Authors:** Ying Tian, Ning Guo, Wen-Yi Wang, Wenming Geng, Li-Chao Jing, Tao Wang, Xiao-Tong Yuan, Zeru Zhu, Yicheng Ma, Hong-Zhang Geng

**Affiliations:** 1 Tianjin Key Laboratory of Advanced Fibers and Energy Storage, School of Material Science and Engineering, Tiangong University, Tianjin, 300387 China; 2 Carbon Star Technology (Tianjin) Co., Ltd., Tianjin, 300382 China

**Keywords:** Materials science, Nanoscience and technology

## Abstract

Graphene oxide (GO), reduced graphene oxide (rGO) and carbon nanotubes (CNTs) have their own advantages in electrical, optical, thermal and mechanical properties. An effective combination of these materials is ideal for preparing transparent conductive films to replace the traditional indium tin oxide films. At present, the preparation conditions of rGO are usually harsh and some of them have toxic effects. In this paper, an SnCl_2_/ethanol solution was selected as the reductant because it requires mild reaction conditions and no harmful products are produced. The whole process of rGO preparation was convenient, fast and environmentally friendly. Then, SEM, XPS, Raman, and XRD were used to verify the high reduction efficiency. CNTs were introduced to improve the film conductive property. The transmittance and sheet resistance were the criteria used to choose the reduction time and the content ratios of GO/CNT. Thanks to the post-treatment of nitric acid, not only the by-product (SnO_2_) and dispersant in the film are removed, but also the doping effect occurs, which are all conducive to reducing the sheet resistances of films. Ultimately, by combining rGO, GO and CNTs, transparent conductive films with a bilayer and three-dimensional structure were prepared, and they exhibited high transmittance and low sheet resistance (58.8 Ω/sq. at 83.45 T%, 47.5 Ω/sq. at 79.07 T%), with corresponding ${{\sigma_{dc} } \mathord{\left/ {\vphantom {{\sigma_{dc} } {\sigma_{opt} }}} \right. \kern-\nulldelimiterspace} {\sigma_{opt} }}$ values of 33.8 and 31.8, respectively. In addition, GO and rGO can modify the surface and reduce the film surface roughness. The transparent conductive films are expected to be used in photoelectric devices.

## Introduction

Graphene is formed of a single layer of carbon atoms with a hexagonal honeycomb structure consisting of two equivalent sub-lattices of carbon atoms bonded together with σ bonds; it is a two-dimensional material. The structure of graphene exhibits good mechanical^1^, electrical^2^, and thermal properties^3^. As a result of these properties, graphene has the potential to be used in the preparation of electrode materials and conductive ink; thus, graphene could be widely used in transparent conductive films and other fields^4–6^.

Following in-depth study of graphene by scientists, demand for graphene for many applications has increased. Finding methods to prepare high-quality graphene at a low cost and in large quantities has become an urgent problem. At present, there are three main methods to prepare graphene: mechanical stripping^7^, chemical vapor deposition (CVD)^8–10^, and redox^11,12^. In 2004, British researchers prepared monolayer graphene by the mechanical peeling method^7^. The graphene prepared by this method is high quality, but its yield is low and the products obtained by each repetition are not always uniform, so it is difficult to use the products in production or life. Graphene also can be produced by CVD. The process of preparing graphene by CVD is simple, and the product quality is good. The area and number of layers of the desired film can be controlled^10^, and large areas can be prepared. However, due to the high temperature during the preparation process, it is difficult to directly prepare it on flexible substrates (such as PET, PEN, etc.) which are commonly used, so transfer operation is required, which is greatly affected by the operators. At present, it is difficult to apply this method to the fabrication of flexible electrodes. Graphene oxide (GO), which can dissolve in water and form uniform and stable films on flexible substrates, is an important raw material for chemical reduction. The main reduction methods of GO are thermal reduction^13,14^ and chemical reduction^15–19^. Thermal reduction refers to the removal of oxygen-containing groups from GO by raising the temperature to hundreds or even thousands of degrees Celsius in the atmosphere of inert gases. The product obtained by this method has few defects and high quality, which is closest to theoretical graphene. However, this method requires harsh reaction conditions and has high requirements for the substrate, which makes it difficult to directly apply to the preparation of flexible transparent conductive films. The reductants needed for chemical reduction include hydroiodic acid (HI)^16^ and hydrazine hydrate (N_2_H_4_·H_2_O)^19^, among others. Although these reductants have low cost, they are highly toxic, pollute the environment, and pose a high risk to operators. Therefore, an environmentally friendly and efficient reducing agent is needed to prepare reduced graphene oxide (rGO).

The measured and theoretical values of graphene conductivity are quite different, mainly because prepared graphene does not have a complete hexagonal honeycomb structure and there are some defects and an imperfect crystal lattice^20^, which is not conducive to electronic transmission. Carbon nanotubes (CNTs) are curled from one or more layers of graphene^21,22^. Graphene itself is an excellent conductive material, so CNTs also have good conductivity^23^. The conductivity of CNTs can be similar to that of metals or semiconductors such as silicon and germanium^24^. The axial conductivity of CNTs is 103 S/cm, and the current density allowed to pass through CNTs is about 109 to 1010 A/cm^2^, which is 1000 times higher than that of copper^25^. Single carbon nanotubes or arrays of several carbon nanotubes exhibit anisotropic conductivity. The conductivity along the diameter direction of the tube is similar to that of metal^26^. A thin CNT grid structure can obtain CNT conductive films with excellent conductivity. This characteristic explains why carbon nanotubes are widely used in transparent conductive film^27^. Therefore, carbon nanotubes can make up for the loss of resistance caused by defects in graphene.

Metal nanowires, metal nanoparticles, conductive polymers and other conductive materials are also available. The salts of silver and copper are usually used to prepare metal nanowires and metal nanoparticles, but the preparation cost is high, and the conductive inks are easily affected by oxygen and moisture in the air, which is easy to deteriorate and seriously affect the conductivity^51,52,55^. The typical representative of conductive polymer is poly(3,4-ethylenedioxythiophene): poly(styrene sulfonate) (PEDOT:PSS). The conductivity of this material is relatively low, even if the conductivity of the new formula is improved, there is still a gap for application. Moreover, it lacks long-term stability under high temperature and humidity^53,54^. Considering the production cost and practical application, carbon nanotubes and graphene are good choices. Carbon materials (including CNTs and graphene) have many advantages for transparent conductive thin films. First, carbon materials come from a wide range of sources. Second, conductive films made of carbon materials are flexible and can be used in various fields^28–31^. Third, they can be prepared conveniently and in large quantities by wet method, and the prepared films have high stability and good performances^32,33^. Therefore, the application prospects of conductive thin films based on CNTs and rGO are strong. SnCl_2_, a non-toxic and harmless powder compound, does not require complex instruments or a strict environment when it is used for GO reduction. The reduction process can be carried out at room temperature and there are no toxic and harmful by-products. The integrity of the films is well-maintained throughout the process, and it is suitable for flexible substrate and industrial large-scale operations. Nitric acid, a strong acid, can effectively remove the by-products from the reduction process and the dispersant needed for the dispersion of carbon nanotubes^40,45,46^. In addition, nitric acid also has N-doping effect on the rGO^42–44^. Through these effects, the sheet resistance of films can be further reduced and the overall conductivity of films can be improved.

In this study, SnCl_2_/ethanol solution, which requires mild reaction conditions and produces no pollution by-products, was selected as the reducing agent. Firstly, the GO films were prepared by spraying method, and the reduction efficiency was verified by SEM, XPS, Raman, and XRD. Then, the reductant was dropped on the transparent conductive films containing carbon nanotubes and graphene oxide, which were also prepared by spraying method. The GO was reduced to rGO. The post-treatment of nitric acid is necessary, which is used to remove by-products and dispersant and bring doping effect. The transmittance and sheet resistance measurements were carried out to determine the appropriate GO/CNT doping ratio, reduction time and processing steps. Ultimately, transparent conductive thin films with a bilayer structure were prepared, which exhibit high transmittance, low surface resistance, and low surface roughness.

## Materials and methods

### Preparation of GO/CNT hybrid solution

High purity (90 wt.%; diameter, 0.8–1.6 nm; length, 5–30 µm) SWCNTs grown by CVD were purchased from the Chengdu Organic Chemicals Co. Ltd., Chengdu, China. GO was synthesized from graphite powder using a modified Hummers’ method. Dodecyl benzene sulfonate (SDBS) was purchased from Aladdin, Shanghai, China and was used as received. CNTs were dispersed in deionized water at a concentration of 1 mg/mL using SDBS (1 wt.%) as a dispersant. The prepared solution was put into a bath sonicator at 90 W for 30 min to dissolve SDBS. The solution was then put into a cellular ultrasound machine at 100 W for 60 min, followed by centrifugation at 8000 rpm for 20 min. The 80% supernatant was taken and the SWCNT solution with a concentration of 1 mg/mL was obtained. This was diluted into 0.2 mg/mL to obtain the required SWCNT solution. The GO solution of 6.8 mg/mL was diluted into 0.2 mg/mL. The hybrid solutions of different proportions were prepared using the above SWCNT and GO solutions according to the mass ratio of solid content in the solutions. Distilled water was added to make all solutions uniform in volume. The specific formulations are listed in Table 1.

**Table 1:** Formula of film-making solutions.

GO/CNT	SWCNTs/mg	GO/mg	Volume of film-making solutions/mL
0.5	1	0.5	15
1.0	1	1	15
1.5	1	1.5	15
2.0	1	2	15

### Preparation of transparent conductive films

**Figure 1:**
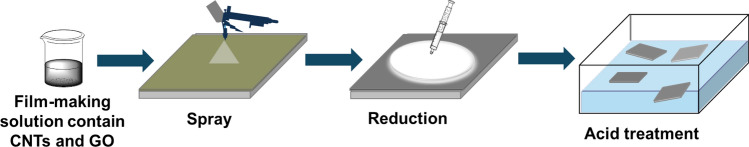
Preparation process of transparent conductive films.

The polyethylene terephthalate (PET) film was cut into 4 × 4 cm squares as the substrate, and then the PET substrate was immersed in ethanol for 30 min to remove impurities. The treated PET was fixed on the heating plate at 120 °C. The above hybrid solutions of GO and CNT were dumped into the air-gun and sprayed onto the surface of the PET substrate with a gas pressure of 0.3 MPa (Fig. 1). After spraying, each GO/CNT transparent conductive film (GO/CNT-TCF) was placed on the plate for about 2 min to evaporate the water from the film. The corresponding transparent conductive films were defined as GO/CNT0.5, GO/CNT1.0, GO/CNT1.5, and GO/CNT2.0.

### Preparation of reducing agent and reduction of GO/CNT-TCFs

SnCl_2_·2H_2_O crystal (4.513 g) was taken and dissolved in 20 mL absolute ethanol. The reducing agent (1 mol/L) was placed in a bath sonicator for 10 min. The principle of reduction is that H^+^ produced by hydrolysis of SnCl_2_ promotes the reduction of GO. The specific principle refers to the following formulae:

1 $$ GO + 2{\text{n}}H^{ + } + 2{\text{ne}}^{ - } \to {\text{rGO}} + {\text{n}}H_{2} O $$

2 $$ S{\text{n}}^{2 + } + 3H_{2} O - 2{\text{e}}^{ - } \to S{\text{n(OH)}}_{{3}}^{ + } + 3H^{ + } $$

3 $$ S{\text{n}}(OH)_{2} - 2e^{ - } \to SnO_{2} + 2H^{ + } $$

The reduction experiment was undertaken as follows (see Fig. 1): the heating plate was adjusted to 60 °C and the GO/CNT-TCFs were placed on it. The prepared SnCl_2_/ethanol solution (about 1 mL for each film) was uniformly dripped onto the surface of GO/CNT-TCFs. When the reaction was conducted for a certain time, the films were cleaned with ethanol and dried; the process was then repeated. The reduction processes were called Reduction 1 and Reduction 2. Ultimately, reduced GO/CNT-TCFs were obtained, named RGO/CNT-TCFs. The RGO/CNT-TCFs corresponding to different GO/CNT ratios were named RGO/CNT0.5, RGO/CNT1.0, RGO/CNT1.5, and RGO/CNT2.0, respectively.

### Acid treatment of RGO/CNT-TCFs

The RGO/CNT-TCFs were immersed in 12 mol/L nitric acid to remove the surfactant, SDBS, for 40 min, followed which they were washed in distilled water three times and dried.

### Preparation of bilayer transparent conductive films

The hybrid solutions with certain proportions of GO and CNTs were divided into two parts on average. For each film, the first part was sprayed and treated following the steps in Fig. 1, while the second part was sprayed on the first layer. The second layers were partly operated according to Fig. 1, while the others were only treated with nitric acid. The corresponding films were named D-RGO/CNT* and S-RGO/CNT*.

### Characterization

The GO solution was dripped onto a copper grid for transmission electron microscope (TEM, TECNAI-20, HITACHI, Japan) analysis^33^. The morphology of the films was observed using a field emission scanning electron microscope (FE-SEM, HITACHI S-4800, HITACHI, Japan) and atomic force microscope (AFM, Icon, BRUKER, America)^32^. Raman spectra were gained to characterize the electronic structure via a DXR Raman microscope with a laser excitation wavelength of 532 nm^32^. The surface changes of functional groups were detected by X-ray photoelectron spectroscopy (XPS, K-alpha, Thermofisher, America )^32^. X-ray diffraction (XRD, D8 DISCOVER, BRUKER, Germany) was used to test the interlayer space. The sheet resistance of the film was measured using a four-point probe (Keithley 2700, Tektronix, America). Transmittance of films was measured using a UV–Vis spectrophotometer at a wavelength of 550 nm^28^.

## Results and discussion

**Figure 2:**
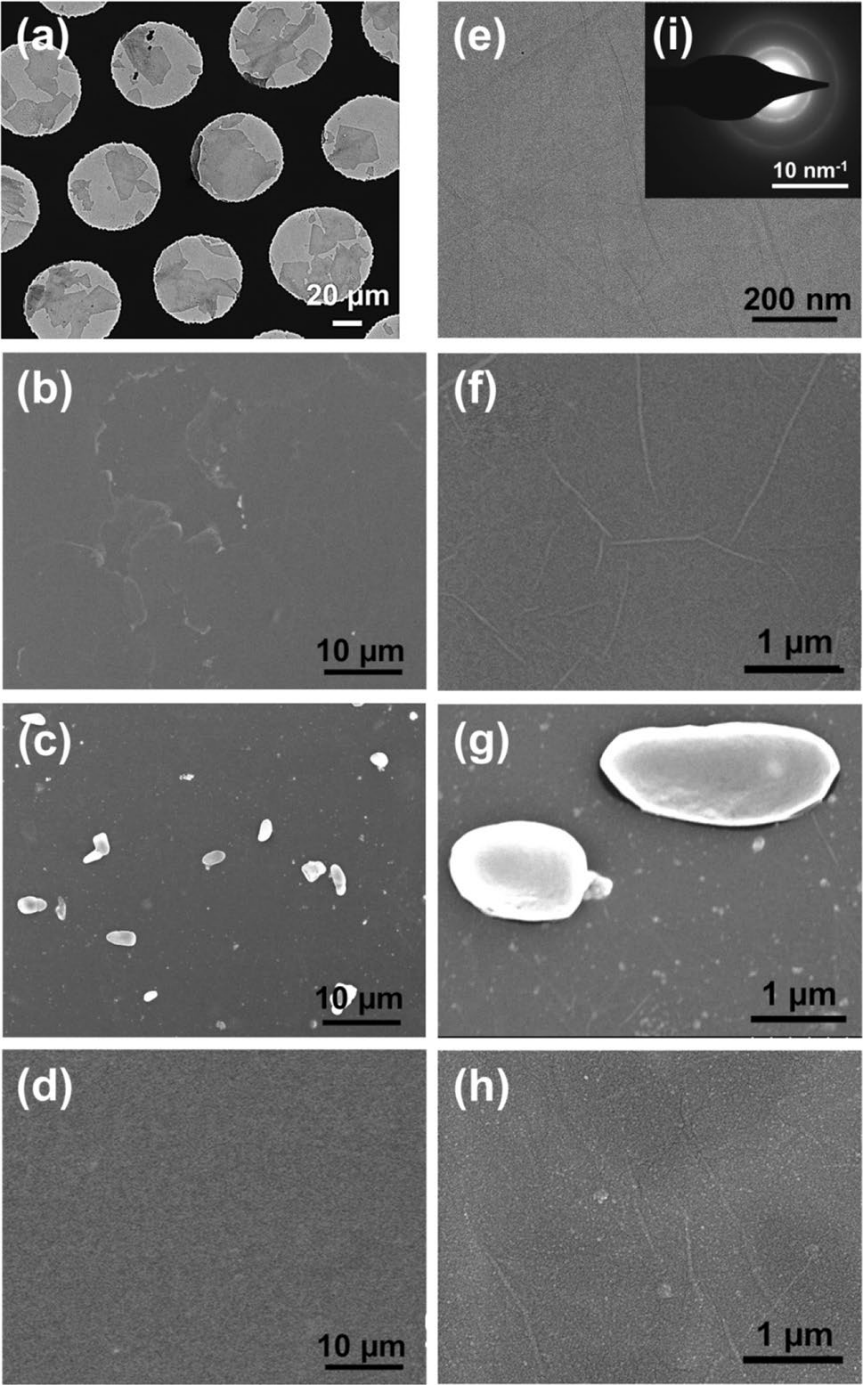
(**a**,**e**) TEM images and (**i**) required diffraction pattern of GO. SEM images of pure GO films (**b**,**f**) without reduction; (**c**,**g**) reduced twice by SnCl_2_ for 10 min; (**d**,**h**) reduced twice by SnCl_2_ for 10 min and treated by acid.

The TEM images of GO and SEM images of films are shown in Fig. 2. The sizes of GO are relatively large, about 10–50 µm (Fig. 2a). The surface of GO is uniform and clean without impurities (Fig. 2e). According to the diffraction pattern (Fig. 2i), the internal structure of GO is uniform. Figure 2b–d shows SEM images of the films that were untreated, reduced for 20 min only, and reduced for 20 min followed by 40 min of acid treatment, named GO films, rGO films, and A-rGO films, respectively. Figure 2f–h are larger images of Fig. 2b–d, respectively. In Fig. 2b, some parts of the film are not dense, while in Fig. 2c, this phenomenon disappears because GO is reduced to rGO. The surface of GO is flat and slightly wrinkled, as shown in Fig. 2f. There are still some wrinkles on the surface of rGO after reduction, and some impurities are accompanied on the surface, as shown in Fig. 2g. The remaining white particles with a uniform size on the surface are by-products—SnO_2_ particles. By comparison with films experiencing reduction only, acid treatment is conducive to removing the SnO_2_ particles on the surface (Fig. 2d,h). This is partly because nitric acid treatment reduces the interaction between SnO_2_ and rGO, which results in the removal of SnO_2_ particles from the films, but there will still be a small number of SnO_2_ particles remaining. In addition, the folds of rGO increase after acid treatment, and the surface is not smooth (Fig. 2h).

**Figure 3:**
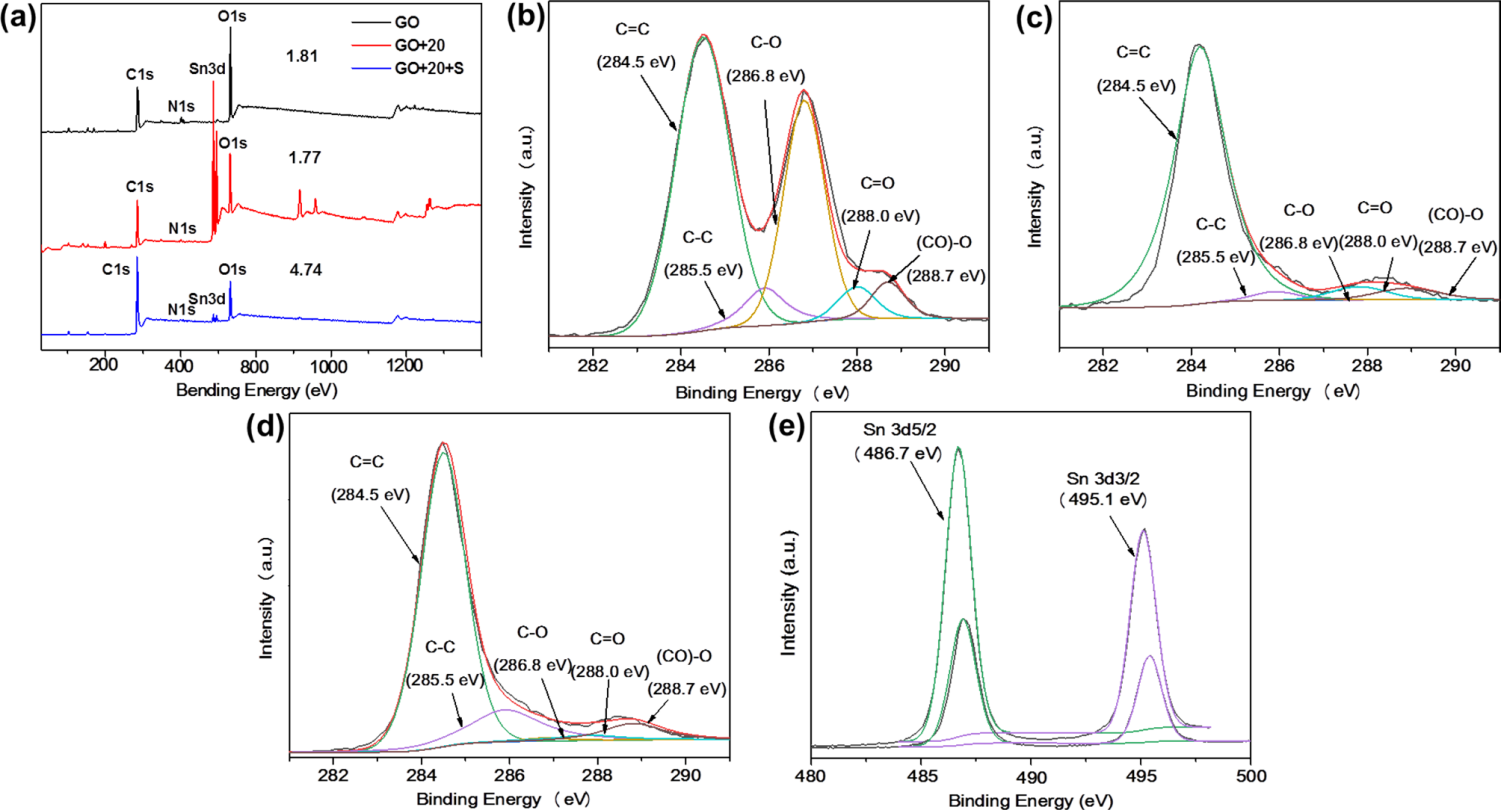
(**a**) XPS spectra of GO films after different treatments; (**b**) C1s spectra of unreduced GO films; (**c**) C1s spectra of GO films reduced twice by SnCl_2_ for 10 min; (**d**) C1s spectra of GO films reduced twice by SnCl_2_ for 10 min and treated by acid; (**e**) Sn_3_d spectra of GO films before and after acid treatment.

Figure 3 shows the XPS spectra of GO films and GO films under different treatment conditions. Figure 3a shows that the C/O atomic ratio increased from 1.91 (pure GO film) to 4.74 (rGO film treated by acid), resulting from an abatement of the oxygen-containing functional groups. Benefiting from the effect of acid, the ratio of C/O increased significantly as the content of SnO_2_ decreased. The reduction of SnCl_2_/ethanol can make the reduced graphene oxide reach such a C/O ratio value, only because some SnO_2_ remains on the surface of the film after reduction, which increases the content of oxygen, so the original C/O ratio is reduced. After treatment with nitric acid, the residual SnO_2_ decreases, thus increasing the C/O ratio, reflecting the true reduction degree of pure oxygen graphene. Therefore, SnCl_2_/ethanol is a good reductant for GO and HNO_3_ can help to remove SnO_2_. Due to the N-doping effect of nitric acid on rGO, the content of N element increased from 2.16 to 2.79% after acid treatment (Fig. 3a). Figure 3b is the C1s peak of pure GO films. The C1s peaks of 284.5, 285.5, 286.8, 288.0, and 288.7 eV correspond to the C=C, C–C, C–O, C=O, and (CO)–O groups, respectively^34,35^, and are in accordance with the existing literature. In comparison with Fig. 3b,c shows the C1s peak of the GO film reduced by SnCl_2_/ethanol and there is a clear distinction between them. The differences are mainly reflected in the intensity of C–O, C=O, and (CO)–O. The intensity of these peaks is obviously reduced, which shows that most oxygen functional groups in GO were removed. The test results provide convincing evidence that SnCl_2_/ethanol, as a non-toxic and harmless reductant, can efficiently reduce GO under general conditions. The C1s spectra of film treated by acid following reduction are shown in Fig. 3d. The peak strength of these oxygen groups was slightly higher compared to Fig. 3c, which indicates that rGO may have oxidized slightly again during nitric acid treatment. Figure 3e shows a comparison of the Sn_3_d peak before and after acid treatment, and reveals that Sn(II) was oxidized to Sn(IV) or SnO_2_ during the reduction process. The height and area of the Sn_3_d peak were markedly reduced after acid treatment, which coincides with the results of Fig. 2c and verifies that HNO_3_ can reduce the content of SnO_2_, leaving some residual.

**Figure 4:**
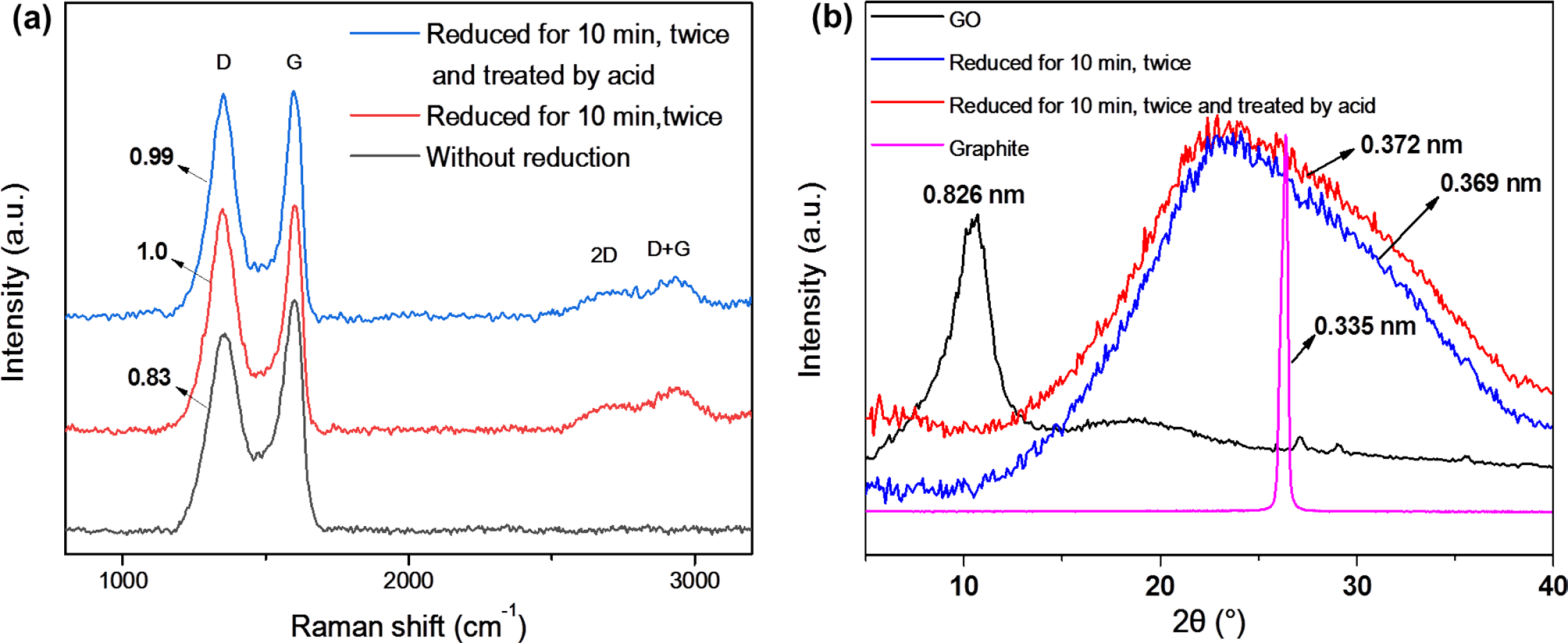
(**a**) Raman spectra (at 532 nm) and (**b**) XRD spectra of GO films under different treatments.

Raman spectra were used to characterize changes to the electronic structure in the reduction and acid process with an excitation laser wavelength of 532 nm (2.33 eV). The G-band (1592 cm^−1^) is the main characteristic peak, which can reflect the ordered degree of graphene. The D-band (1351 cm^−1^) is generally used to characterize the defects of graphene. The intensity ratio of the D-band to the G-band (*I*_*D*_*/I*_*G*_) is used to characterize the reduction degree of graphene^36^. A higher value of *I*_*D*_*/I*_*G*_ means more oxygen-containing functional groups are removed from GO films, indicating high reduction degree of graphene. Figure 4a shows that the value of *I*_*D*_*/I*_*G*_ increases from 0.83 to 1.0 and then changes to 0.99. The three values from the beginning to the end correspond to GO films, rGO films, and A-rGO films, respectively. The increase can be attributed to the recovery of the sp2-bonded carbon atoms in a 2-dimensional hexagonal carbon lattice, showing the reduction of carbon–oxygen bonds^37^. The *I*_*D*_*/I*_*G*_ value decreased slightly to 0.99 because acid treatment can destroy sp2-bonded carbon atoms and add the number of carbon–oxygen bonds to some extent. The appearance of a 2D-band near 2690 cm^−1^ shows that the rGO had some defects, becoming a graphite structure, while the D + G-band near 2930 cm^−1^ are usually considered to have been caused by defects^38^. XRD spectra were used to characterize the reduction degree of GO with Kα1 of copper target (0.154 nm) and scanning range of 4°–40°. Figure 4b shows the XRD spectra of graphite and film samples including GO, rGO, and A-rGO. The basic criterion for comparison is graphite, which has perfect sp2-bonded carbon atoms. The (002) diffraction angle of graphite was 26.6°. According to the Bragg formula (4), the interlayer space was calculated to be 0.335 nm.

4 $$ 2{\text{dsin}}\theta = \lambda $$

By comparison with graphite, GO has a small diffraction angle (2*θ* = 10.7°) and a large interlayer space (0.826 nm), which is due to the grafting of carboxyl and hydroxyl groups between the graphene layers after oxidation^39^. After reduction by the SnCl_2_/ethanol solution, the oxygen groups decrease, sp2-bonded carbon atoms reconstruct, and graphene sheets become close by the π–π stacking effect. This leads to the graphene sheets having a diffraction angle of 24.1°, corresponding to the interlayer spacing of 0.369 nm, and showing that the conjugated graphene network is re-established. Moreover, the single diffraction peak of rGO shows that the crystal structure after reduction is relatively uniform. It is safe to conclude that the SnCl_2_/ethanol solution, as a non-toxic and mild reductant, can effectively reduce graphene oxide under general conditions. Significantly, there are still differences between rGO and graphene, for example, in terms of the position of diffraction peaks. Therefore, it is difficult to completely eliminate oxygen-containing groups of GO through chemical reduction. Perhaps this is the reason why the new layer spacing was larger than the pure graphite. After nitric acid treatment, the diffraction angle slightly reduced (2*θ* = 23.9°) and the interlayer spacing increased to 0.372 nm. Plainly, nitric acid can cause partial rGO to be oxidized again, but the degree of oxidation is very low.

**Figure 5:**
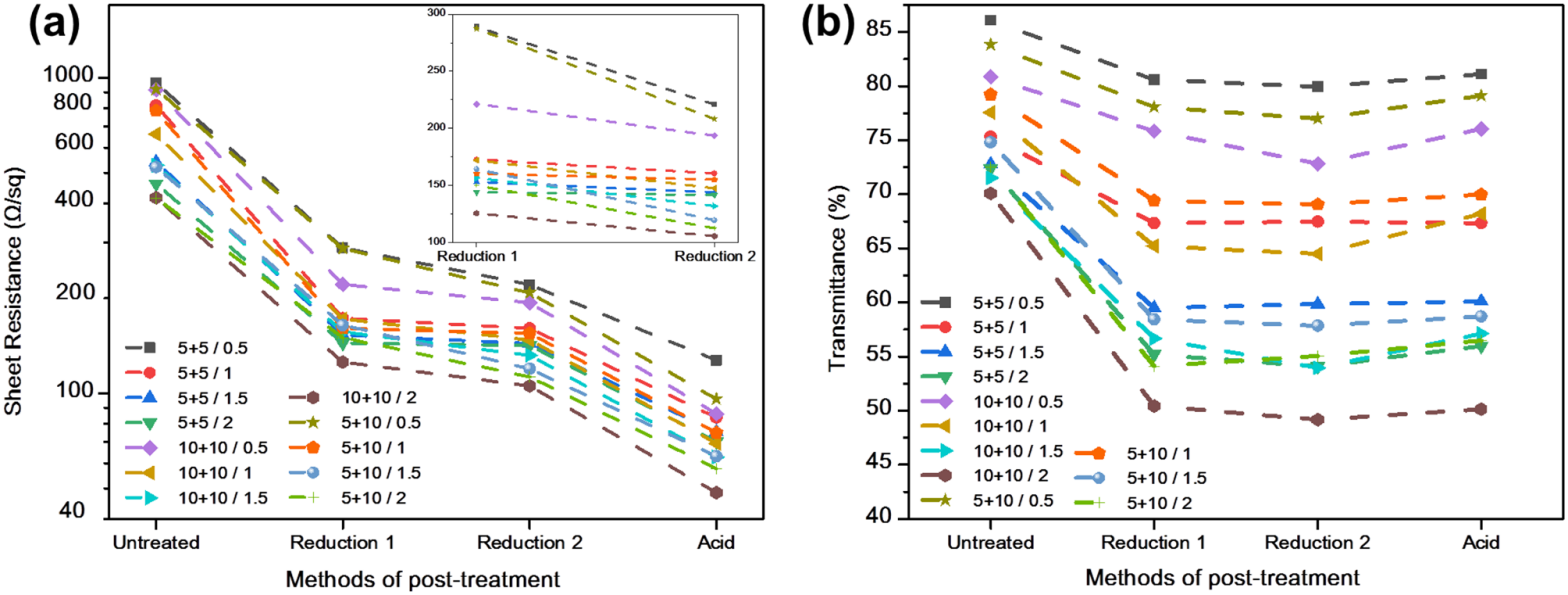
Change in (**a**) sheet resistance and (**b**) transmittance with different RGO-CNT films consisting of different GO/CNT ratios and experiencing different processing times and processing steps.

Figure 5a shows the sheet resistance curves of transparent conductive films prepared by spraying a GO/CNT mixed solution at a ratio of 0.5, 1.0, 1.5, and 2.0 under three different processing times and different processing steps. First, analysis was carried out under conditions with the same processing time. Figure 5a shows that with the increase of GO doping content, the sheet resistances of the transparent conductive films all show a downward trend under each processing step. Carbon nanotubes are one-dimensional materials, while graphene oxide and reduced graphene oxide are two-dimensional materials, so a combination of these three materials can contribute to form a three-dimensional conductive network structure and, with the increase of GO content, the network tends to be perfect. This may partly explain why the sheet resistance decreased. The increase of GO inevitably led to the decrease of transmittance, so there was no need to test a larger proportion of GO. Second, an analysis of reduction time was carried out with fixed GO content. As the reduction time increased, the sheet resistance decreased. However, the longer the reduction time was, the smaller the change of sheet resistance was, that is to say, the difference between the three sheet resistance values of GO/CNT films with same ratio after 10, 15 and 20 min of reduction was reduced, especially the sheet resistance values after 15 min and 20 min reduction. This phenomenon suggests that a reduction time of 20 min is sufficient. Finally, the influence of each processing stage on sheet resistance was analyzed. After the first reduction (Reduction 1), the sheet resistance of GO/CNT transparent conductive films dropped. Among them, GO/CNT0.5 films reduced for 10 min and GO/CNT1.0 films reduced for 10 min with a greater downward trend. After the second reduction (Reduction 2), the overall reduction slowed down. After the nitric acid treatment, sheet resistance reduced again. The reason why GO/CNT0.5 and GO/CNT1.0 decreased more than the other ratios suggests that the structure of the three-dimensional conductive network was not perfect. In this case, the reducing agent can penetrate into the inner layer of the thin films through the gap between GO and CNT. Therefore, the sheet resistance of thin films decreases more. At other ratios, the surface of the thin film was basically covered by GO, so it was more difficult for the reducing agent to penetrate deeply. Accordingly, there will be more GO which is not been reduced sufficiently in this case. There are three reasons why nitric acid caused a reduction in the sheet resistance. First, the removal of SDBS may explain this, as SDBS is a dispersant, that is, a non-conductive substance. H^+^ provided by nitric acid is combined with sulfonic acid group in SDBS, which increases the hydrophilicity of SDBS, so most of the remaining SDBS can be washed away^40,45,46^. Second, it may be due to the removal of the by-product, SnO_2_. SnO_2_ particles generated in this experiment had a large particle size, which would increase the sheet resistance. Under certain conditions, nitric acid can remove SDBS and SnO_2_ particles, which would cause the sheet resistances to be reduced. Third, nitrogen doping will occur when rGO is immersed in nitric acid, which can improve the conductivity of rGO^42–44^, thus reducing the sheet resistance of the whole film. The standard deviations of sheet resistances of different proportions in different stages are 100(GO/CNT: 0.5,1), 50(GO/CNT: 1.5, 2) (Untreated); 50(GO/CNT: 0.5), 20(GO/CNT: 1,1.5, 2) (Reduction 1); 25(GO/CNT: 0.5, 1, 1.5, 2) (Reduction 2); 20(GO/CNT: 0.5,1,1.5, 2) (Acid). Figure 5b shows the transmittance curves of transparent conductive films prepared by spraying the GO/CNT mixed solution with a ratio of 0.5, 1.0, 1.5, and 2.0 under three different processing times and different processing steps. Figure 5b shows that with an increase of GO doping, the overall transmittance decreases. In addition, with the same GO content, the longer the reduction time, the lower the transmittance. This is because GO and rGO have the function of shading light to a certain extent. After the first reduction (Reduction 1), the transmittance of GO/CNT2.0 films reduced for 10 min decreased the most. This may be due to the longer reduction time and higher GO content (transmittance is lower when more graphene is produced by reduction). After the second reduction (Reduction 2), the transmittance of all films decreased to a lesser extent, which may be due to the fact that the first reduction was sufficient and the second reduction could not produce a large amount of graphene, so transmittance changed less. Thus, transmittance results corroborate the sheet resistance results. After acid treatment, the transmittance increased slightly; this could be due to the removal of SDBS and SnO_2_ grains. Without the shielding of these particles, light can pass through the film, so that the light transmission is slightly improved. The standard deviations of transmittance of different proportions in different stages are 4 (GO/CNT: 0.5,1,1.5,2) (Untreated); 4 (GO/CNT: 0.5,1,1.5,2) (Reduction 1); 4 (GO/CNT: 0.5,1,1.5,2) (Reduction 2); 4 (GO/CNT: 0.5,1,1.5, 2) (Acid).

**Figure 6:**
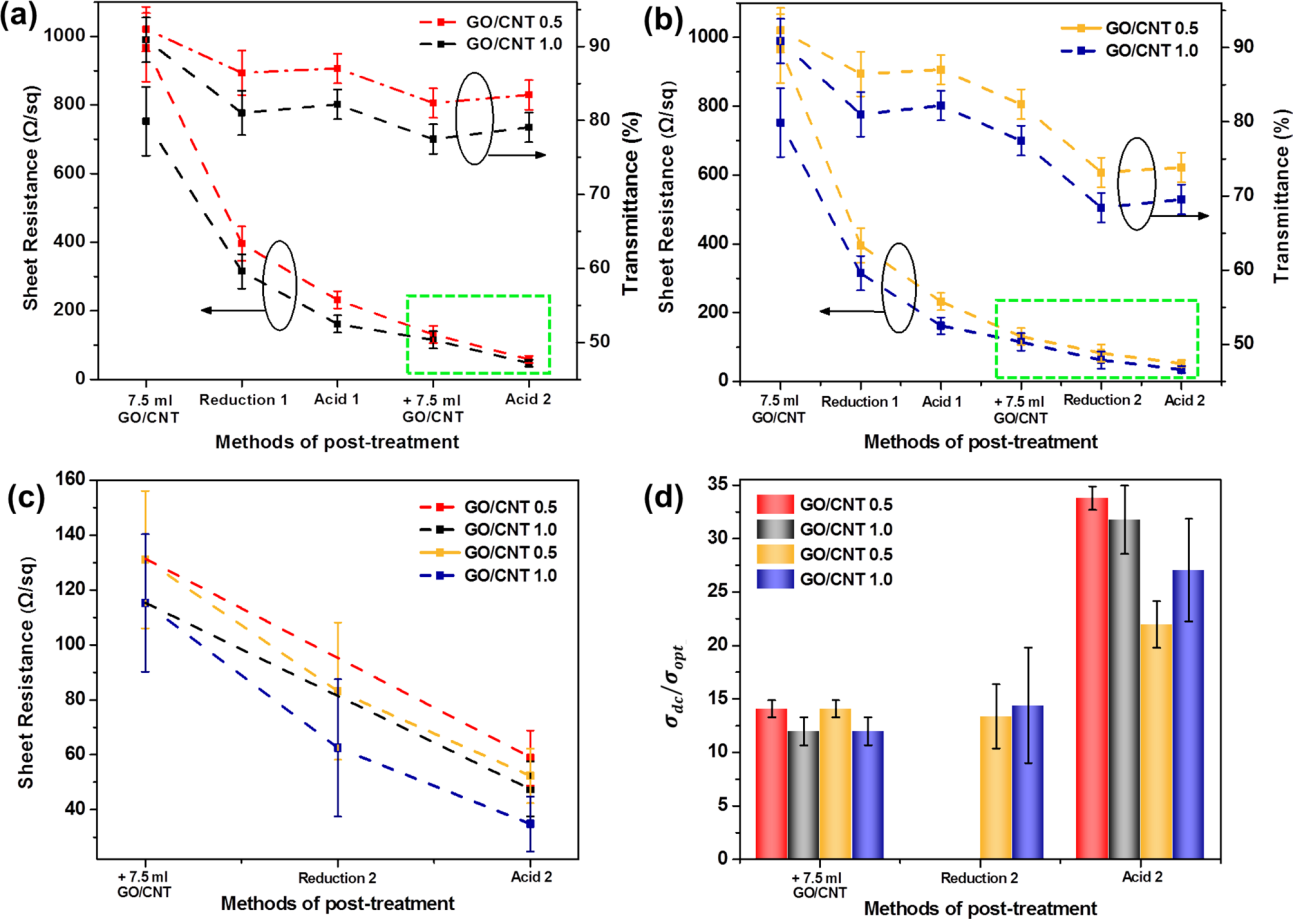
Sheet resistance and transmittance of bilayer films after treatments: (**a**) S-RGO/CNT0.5 and S-RGO/CNT1.0, (**b**) D-RGO/CNT0.5 and D-RGO/CNT1.0. Comparison of (**c**) sheet resistance values and (**d**) ${{\sigma_{dc} } \mathord{\left/ {\vphantom {{\sigma_{dc} } {\sigma_{opt} }}} \right. \kern-\nulldelimiterspace} {\sigma_{opt} }}$ values shown in green boxes of (**a**,**b,** respectively).

Figure 6 shows schematic diagrams of the sheet resistance and transmittance of the transparent conductive bilayer films after different treatments. Figure 6a,b shows that the trend of sheet resistance and transmittance before spraying the second layer was the same as shown in Fig. 5. After spraying with 7.5 mL of GO/CNT mixed solution, the sheet resistance decreased again. The reducing trend could be attributed to a parallel structure and a more perfect three-dimensional network structure; the former double-layer structure is equivalent to two resistors in parallel, which will reduce the total sheet resistance, while for the latter, the two layers of GO/CNT can form a more perfect three-dimensional structure, which increases the electronic transmission path and reduces the sheet resistance. Moreover, the transmittances all decreased. After acid treatment without reduction, the sheet resistance continued to decrease and transmittance increased slightly, as shown in the green box in Fig. 6a. The reasons for decreasing of sheet resistance are three: the removal of SDBS, the removal of SnO_2_ and the doping of GO, as explained in Fig. 5. As for light transmission, the light will not be blocked due to the removal of SDBS and SnO_2_ particles, so the transmittance of the film can be improved, but the improvement is limited. Thus, transparent conductive films with high transmittance and low sheet resistance can be created, with the best properties obtained being 58.8 Ω/sq. at 83.45 T% and 47.5 Ω/sq. at 79.07 T%. After reduction and acid treatment, the sheet resistance maintained a continuous downward trend, and transmittance decreased first but then increased (green box in Fig. 6b); the best properties obtained were 52.3 Ω/sq. at 73.86 T% and 34.9 Ω/sq. at 69.52 T%. Through the comparison of the two experiments (Fig. 6c), the values of sheet resistance can be reduced by adding a reduction step in the second layer, but the reduction amplitude is not very large, and the transmittance will be reduced by about 10%. The ratio of direct current conductivity to optical conductivity (${{\sigma_{dc} } \mathord{\left/ {\vphantom {{\sigma_{dc} } {\sigma_{opt} }}} \right. \kern-\nulldelimiterspace} {\sigma_{opt} }}$) is considered an index with which to evaluate the comprehensive performance of transmittance and sheet resistance for transparent conductive films. An approximate relationship between sheet resistance, optical transmittance, and ${{\sigma_{dc} } \mathord{\left/ {\vphantom {{\sigma_{dc} } {\sigma_{opt} }}} \right. \kern-\nulldelimiterspace} {\sigma_{opt} }}$ is given by the following formula^4,41^:

5 $$ T = \left[ {1 + \frac{188(\Omega )}{{R_{S} }}\frac{{\sigma_{{{\text{opt}}}} }}{{\sigma_{dc} }}} \right]^{ - 2} $$

6 $$ \frac{{\sigma_{dc} }}{{\sigma_{opt} }} = \left[ {\frac{188(\Omega )}{{R_{s} }}\frac{\sqrt T }{{1 - \sqrt T }}} \right] $$

The values of ${{\sigma_{dc} } \mathord{\left/ {\vphantom {{\sigma_{dc} } {\sigma_{opt} }}} \right. \kern-\nulldelimiterspace} {\sigma_{opt} }}$ corresponding to the points of green boxes in Fig. 6a,b are shown in Fig. 6d. Although the reduction treatment of the second layer reduces the sheet resistance by a small margin, the transmittance exhibit up to a 10% reduction. Therefore, the values of ${{\sigma_{dc} } \mathord{\left/ {\vphantom {{\sigma_{dc} } {\sigma_{opt} }}} \right. \kern-\nulldelimiterspace} {\sigma_{opt} }}$ do not show significant upward trends and may even exhibit a downward trend. While a subsequent acid treatment can improve the overall performance to some extent, it is still worse than the performance of the films without reduction. Considering transmittance and sheet resistance, it is more appropriate not to add the reduction step in the second layer.

**Figure 7:**
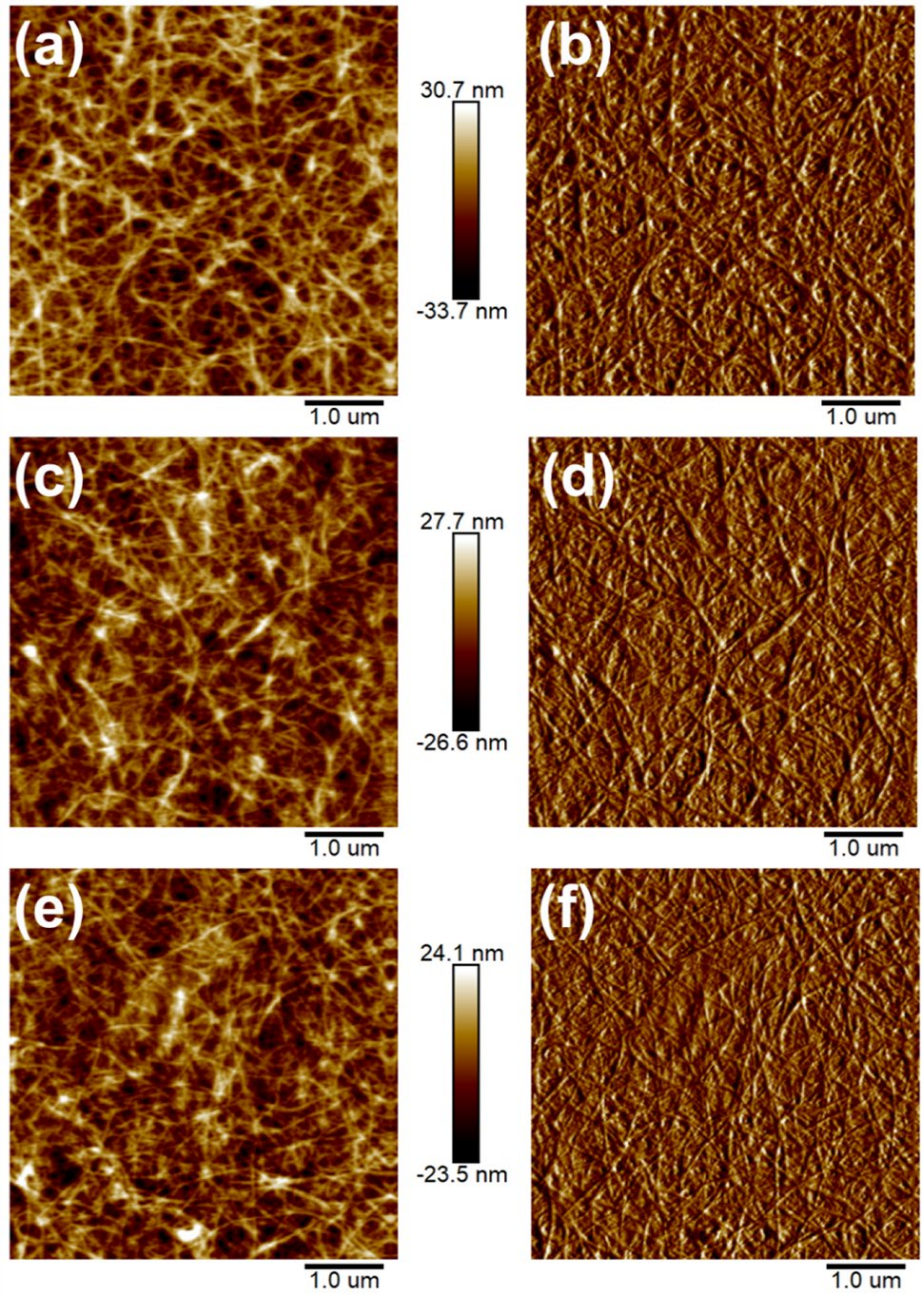
AFM height and peak force error diagrams: (**a**,**b**) Pure CNT films; (**c**,**d**) S-RGO/CNT0.5 films; (**e**,**f**) S-RGO/CNT1.0 films.

The AFM images about surface morphology of pure CNT films, S-RGO/CNT0.5 films and S-RGO/CNT1.0 films are shown in Fig. 7. As shown in Fig. 7a, the root mean square (rms) roughness of transparent conductive films prepared by CNT bundles is relatively high (9.24 nm). This is because CNTs, a one-dimensional material, form films by overlapping with each other, which not only forms an inhomogeneous surface, but also can introduce defects that leads to undesirable outcomes such as tip discharge. GO and rGO are two-dimensional materials, which suggests that the surface roughness of transparent conductive films doped with GO and rGO can be reduced to a certain extent. The root mean square (rms) roughness of S-RGO/CNT0.5 films (Fig. 7b) and S-RGO/CNT1.0 films (Fig. 7c) were 7.63 nm and 6.74 nm, respectively. There is a good fit between the speculation and the results. This implies that it is advantageous to use hybrid films as anodes to fabricate organic light emitting diode (OLED) devices which have high requirements with regard to the surface roughness of anodes.

**Figure 8:**
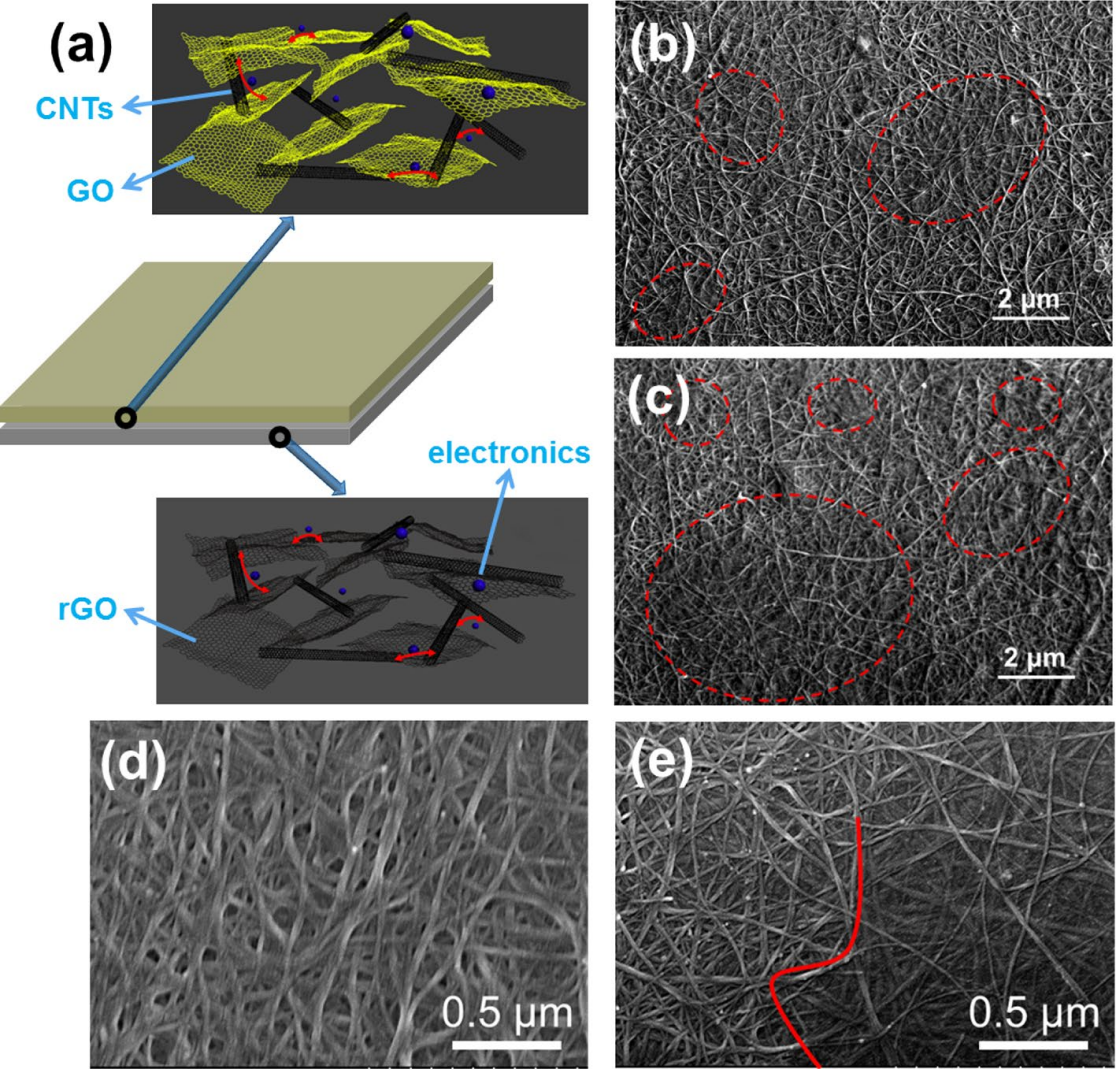
(**a**) Structural schematic map of S-RGO/CNT* films containing CNTs, GO, and rGO. SEM pictures of films with a bilayer structure: (**b**) S-RGO/CNT0.5; (**c**,**e**) S-RGO/CNT1.0. (**d**) pure CNT film.

Figure 8a,b,c,e shows the schematic map and scanning electron microscopy (SEM) pictures of S-RGO/CNT* films. The red circles in Fig. 8b,c and right side of the red line in Fig. 8e represent where GO exists. Figure 8d shows a pure CNT film. Figure 8a shows that the GO, rGO, and carbon nanotubes mingle with each other. For electrons, carbon nanotubes only have one-dimensional conduction path. When carbon nanotubes form a network structure, they have two-dimensional conduction paths. When a certain thickness of carbon nanotubes is sprayed to make them into thin films, the distribution of CNTs is non oriented and some electrons can be transferred vertically, so they have three-dimensional conductive paths, as shown in Fig. 8d. Graphene oxide itself has a two-dimensional conductive path. When GO and CNTs are mixed and sprayed to form the second layer, three-dimensional structure will be formed, as shown in Fig. 8e. The process of reducing GO to rGO does not affect the formed three-dimensional conductive path of the first layer. Therefore, the internal structure of the first layer is also a three-dimensional structure. Between the two layers, the film is not completely flat, there will be some bumps and some electrons are transferred vertically. In the horizontal plane, electrons can be transferred on the CNTs, GO and rGO. Due to the non directional distribution of CNTs and the bulges on the surface of the film, some electrons in the whole film, including between the two layers, can be transferred vertically. The whole film has a double layer and three-dimensional structure and this structure can shuttle electrons (blue balls) back and forth. In Fig. 8e, the red line is the dividing line between the area with GO and the area without GO. The area covered by GO is gray and the network of CNT is fuzzy. The area without GO coverage is bright and the network of CNT is clear. According to this method to estimate the coverage area of GO, Fig. 8b shows that ~ 15% of the area is covered by GO, whereas in Fig. 8c ~ 40% of the area is covered in GO. The amount of GO and rGO in Fig. 8c is greater than in Fig. 8b, which is conducive to forming a more perfect three-dimensional conductive network in the films, thus reducing sheet resistance. Meanwhile, flat two-dimensional GO sheets can reduce the surface roughness of films, increase the hole injection rate and improve the performance of OLED devices in the future.

**Figure 9:**
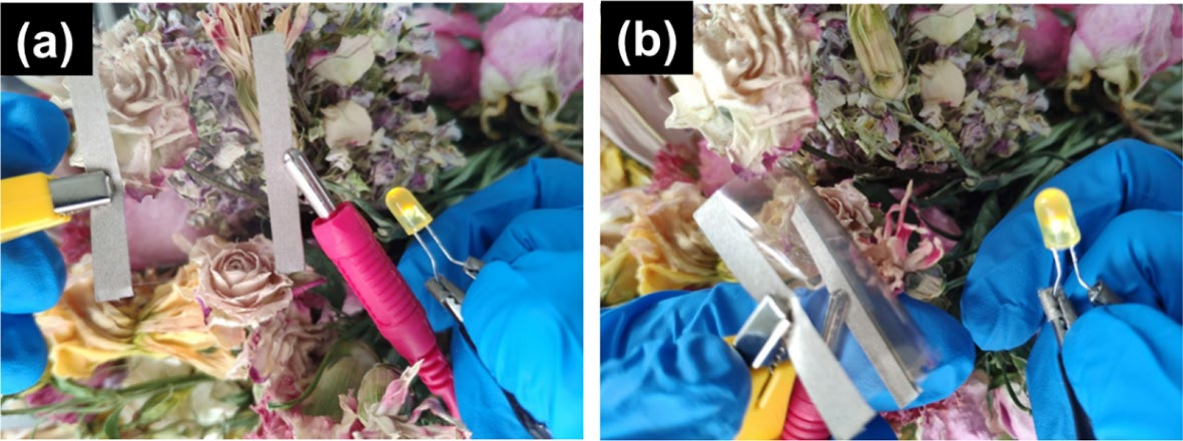
Demonstration of the flexible transparent film of S-RGO/CNT1.0 for lighting LED: (**a**) film without bending, (**b**) film in bending state.

Figure 9 are demonstration of the flexible transparent film of S-RGO/CNT1.0 for lighting LED. As shown in the pictures, the film is transparent and conductive (Fig. 9a), and even in bending state, it can still maintain good electrical conductivity (Fig. 9b). In this paper, graphene was prepared by reduction and then compounded with CNTs. Graphene/metal^48,49^ or graphene/metal oxide^47^ composite films have also been widely studied. Graphene can be compounded with silver, copper and other metals, the latter usually appears in the form of nanowires, metal grids and so on, which also brings the problem of large surface roughness. Because the metal is easy to be oxidized, the film is unstable. Graphene can improve the stability and surface roughness to some extent. This kind of films usually have transmittance above 80%, very low sheet resistance, and good mechanical properties. Considering the transfer of graphene, the preparation of this kind of films is not easy^50^. Graphene can be compounded with metal oxides such as ITO. Due to the poor mechanical properties of ITO, although graphene can improve the mechanical properties of ITO, the overall mechanical properties is still poor. This kind of films can obtain transmittance more than 80%, low sheet resistance and low surface roughness^47^. Compared with these two kinds of films, the films prepared in this study have the same transmittance, low sheet resistance, good mechanical properties, low surface roughness, good stability and simple preparation process.

## Conclusions

In this paper, an SnCl_2_/ethanol solution was used as the reducing agent to reduce pure GO. Compared with the traditional method, the whole process of rGO preparation is fast, efficient, environmentally friendly and does not generate harmful by-products. The results of XPS, Raman, and XRD show that the reagent had good reduction efficiency, and the ratio of C/O achieved was 4.74. Then, the SnCl_2_/ethanol solution and nitric acid were used to reduce and soak graphene oxide doped carbon nanotube transparent conductive films, respectively. The results showed that the appropriate doping ratios (GO/CNT) were 0.5 and 1.0, and the optimum reduction time was set at 20 min. The combination of rGO and CNTs aims to complement each other, that is, improving the sheet resistance and surface roughness at the same time. The use of nitric acid has multiple effects, that is, eliminating impurities and introducing doping effect. To further improve the performance of films, bilayer films were prepared on the basis of the above analyses. The first layer was reduced twice by SnCl_2_/ethanol solution for 10 min and then underwent an nitric acid treatment. The second layer was treated by nitric acid only. The optimal result was 58.8 Ω/sq. at 83.45 T% and 47.5 Ω/sq. at 79.07 T%, and the corresponding values of ${{\sigma_{dc} } \mathord{\left/ {\vphantom {{\sigma_{dc} } {\sigma_{opt} }}} \right. \kern-\nulldelimiterspace} {\sigma_{opt} }}$ were 33.8 and 31.8. The bilayer structure makes the reduction, impurity removal and doping effect more fully. The reduction of GO to rGO can effectively improve the conductivity. The transmittance and sheet resistance properties of the films are improved by the introduction of CNT, the proportion adjustment of each component and the post-treatment. In addition, the existence of GO and rGO is beneficial to obtain the film with lower surface roughness. The results showed that the combination of GO, rGO, and CNTs not only obtained films with excellent photoelectric properties, but also effectively reduced the surface roughness of films. The transparent conductive films are expected to be used in the future in flexible photoelectric devices.

## Data Availability

The datasets generated during and analysed during the current study are available from the corresponding author on reasonable request.
